# The Association between Depression, Anxiety, and Thyroid Disease: A UK Biobank Prospective Cohort Study

**DOI:** 10.1155/2024/8000359

**Published:** 2024-07-04

**Authors:** Tianqing Fan, Xuerong Luo, Xuting Li, Yanmei Shen, Jiansong Zhou

**Affiliations:** ^1^Department of Psychiatry, National Clinical Research Center for Mental Disorders, and National Center for Mental Disorders, The Second Xiangya Hospital of Central South University, Changsha 410011, Hunan, China; ^2^Clinical Nursing Teaching and Research Section, The Second Xiangya Hospital, Central South University, Changsha, China; ^3^Department of Thoracic Surgery, The Second Xiangya Hospital, Central South University, Changsha, Hunan, China

## Abstract

**Background:**

There is a lack of comprehensive data regarding the association of depression and anxiety for the subsequent risk of thyroid disease.

**Aim:**

To examine this prospective association between depression and anxiety and the risk of potential thyroid diseases, a large prospective study was conducted using data from UK Biobank.

**Materials and Methods:**

Depression and anxiety were measured through self-reported Patient Health Questionnaire-4 (PHQ-4) at baseline, thyroid disease was diagnosed by corresponding hospital data documented through International Classification of Diseases (ICD)-10 codes. Cox proportional hazard models and restricted cubic splines were employed to analyze the associations.

**Results:**

In a 13-year follow-up cohort study involving 349,993 participants, 9,877 (2.82%) individuals developed thyroid disease. Significant associations were observed between depression, anxiety, and risk of both hypothyroidism and hyperthyroidism among all participants, the adjusted hazard ratios (HRs) for PHQ scores were as follows: mild 1.27 (95% CI, 1.20–1.36), moderate 1.33 (95% CI, 1.17–1.51), and severe 1.56 (95% CI, 1.31–1.85). For hyperthyroidism, the HRs were mild 1.19 (95% CI, 1.03–1.38), moderate 1.43 (95% CI, 1.08–1.90), and severe 1.84 (95% CI, 1.28–2.66). The adjusted HRs indicating the association between depression, anxiety, and hypothyroidism remained significant for both males and females. The relationship between PHQ score and the risk of thyroid disease shows linear in restricted cubic spline.

**Conclusion:**

Depression and anxiety exposure is associated with subsequent risk of thyroid diseases, the more severe the depression and anxiety, the greater the associated risk. Therefore, any level of depression and anxiety should be taken into consideration in thyroid disease risk prediction and prevention.

## 1. Introduction

Thyroid disorder is a broad-spectrum series disfunction of thyroid hormones and morphological changes, which is common with a prevalence of 1.3% in a global context, and the annual incidence rate was 51 cases per 100,000 individuals [1]. Thyroid hormones exert influence across every phase of our lives, playing a pivotal role in regulating growth and metabolism [2] with sex differences [3]. Its dysfunctionality is often associated with various illnesses, such as increased risk of psychiatric illnesses, especially for anxiety and depression [4]. Though nowadays awareness regarding thyroid illnesses has been elevated as well as the measurement of thyroid hormones through sensitive laboratory tests allows for acute diagnosis of thyroid disorders [5], several of them did not receive timely diagnosis and treatment due to the complicated clinical manifestations, therefore leading to severe consequences [6, 7].

Depression and anxiety are common mental health illness that not only significantly impairs individuals' social and psychological functioning but also decrease their quality of life, with a prevalence of 6% [8] and 18% [9], respectively. Studies have indicated a frequent comorbidity between depression, anxiety, and thyroid disorders, including hyperthyroidism [10], hypothyroidism [11], and autoimmune thyroid diseases [12]. For example, most individuals with depression are found to have thyroid function abnormalities [13], one recent study showed elevated thyroid stimulating hormone (TSH) in 1,718 first-episode and drug-naïve major depressive disorder patients [14], another study conducted in 9,471 participants indicated that depressive symptom is cross-sectionally associated with lower TSH levels and higher free tetraiodothyronine (fT4) levels [15]. Besides, individuals with depression are frequently observed to have clinical or subclinical hypothyroidism [16]. Thyroid hormones have been frequently utilized to enhance the effectiveness of antidepressant treatments [11]. Another study revealed abnormal thyroid function in individuals with anxiety disorder [17]), and antithyroglobulin antibody were found to predict anxiety symptoms in female patients [18]. Conversely, individuals with thyroid disorders are also prone to comorbid with depression and anxiety [19, 20, 21]. For example, lifetime prevalence of depression is significantly higher in patients with subclinical thyroid dysfunction [22]. Individuals with hyperthyroidism had a higher chance of being diagnosed with clinical depression compared to those with normal thyroid function [23]. A recent cohort study included 29,034 patients reported that low TSH was associated with an increased likelihood of depression [24]. Previous research has reported associations between depression, anxiety, and their symptoms often overlap. For instance, depression shares several symptoms with hypothyroidism, such as persistent low energy, lack of concentration, memory impairment, sleep disturbances, and absence of interest [25, 26]. There are also similar symptoms between anxiety and hyperthyroidism, such as nervousness, restlessness, irritability, palpitations, tremors, and insomnia [27, 28]. Though comorbidities are widely seen, causation relationship remains unclear.

In conclusion, to our knowledge, there are only a few studies examining the association between anxiety, depression, and the development of thyroid disorder, with majority of them exploring the influence of thyroid dysfunction on depression and anxiety, there is a lack of comprehensive data regarding the association of depression and anxiety for the risk of thyroid disorders. We address this limitation by employing a cohort study design with data from the UK Biobank, to examine the prospective association between depression, anxiety, and the risk of potential thyroid diseases. We hypothesized that both anxiety and depression would be associated with thyroid disease among participants in the UK Biobank.

## 2. Materials and Methods

### 2.1. Study Design and Participants

The UK Biobank is a large population-based prospective cohort study designed to investigate the determinants of illnesses in middle-aged and elderly individuals. Between 2006 and 2010, data at baseline were collected from over 500,000 participants aged 40–69 years old from 22 different assessment centers in England, Scotland, and Wales [29]. Participants need to register with a general practitioner and reside within 25 miles of an assessment center to be eligible for participation. During the baseline assessment, detailed information on demographics, lifestyle, and medical history was collected from all recruited individuals using touchscreen questionnaires. Participants also provided information about medical diagnoses through computer-assisted personal interviews conducted by trained interviewers. Hospital Episode Statistics (HES) data have been linked to the UK Biobank. HES is a national dataset for England containing ICD-10 diagnoses for hospital admissions. All participants provided informed consent, and ethical approval was granted by the UK Biobank through the UK National Health Service's National Research Ethics Services (16/NW/0274).

In the current study, the sample was selected based on those who completed the baseline assessment (*N* = 502,379) between 2006 and 2010, it was followed from the baseline assessment until the end of the study period in July 2023, resulting in a follow-up period of 1–13 years. Participants diagnosed with any thyroid disease (e.g., hypothyroidism, hyperthyroidism, nontoxic goiter (NTG), and thyroiditis) were excluded (*n* = 33,614), as well as with missing items in exposure and outcome (*n* = 118,772). Finally, 349,993 participants were included, those with depression and anxiety represented the exposed group, and others made up comparison group.

### 2.2. Outcome

The primary outcome measure was the thyroid disease, defined as hypothyroidism, hyperthyroidism, NTG, and thyroiditis in this study. Utilizing inpatient medical records from a hospital stay, the clinical firstly diagnosis of thyroid disorder was documented through International Classification of Diseases (ICD)-10 [30] codes (UK Biobank data field 41202). Specifically, this includes hypothyroidism (ICD-10 codes: E02 and E03), hyperthyroidism (ICD-10 code: E05), NTG (ICD-10 code: E04), and thyroiditis (ICD-10 code: E06). Subjects with any form of thyroid disorder at any point before baseline were excluded from the analysis.

### 2.3. Exposure

Self-reported Patient Health Questionnaire-4 (PHQ-4) were used to assess potential depression and anxiety symptoms. The baseline measurement of PHQ-4 [31] in the UK Biobank study has been previously elaborated. The PHQ-4 consists of two subscales, namely the PHQ-2 and the Generalized Anxiety Disorder screening tool (GAD)-2. These subscales are derived from the diagnostic and statistical manual of mental disorders-5 (DSM-5) criteria for diagnosing depression and GAD separately, demonstrating reasonable specificity and sensitivity for symptoms. It has a 3 Linkert score for each question, and based on the original classification standard, the divisions include normal (0–2), mild (3–5), moderate (6–8), and severe (9–12) [32]. The instrument demonstrates strong internal consistency [33], with a Cronbach's alpha of 0.80 [34].

### 2.4. Covariates

By conducting a literature review, we incorporated factors associated with depression and thyroid disorders as confounding variables into the analysis. These variables included self-reported age, sex (female vs. male, assigned at birth), education which separated into five stages, BMI, living in a socioeconomically deprived area (quintiles based on Townsend deprivation indices), and self-reported data on the age at which current smokers started to smoke or the age ex-smokers stopped smoking were used to group participants into current smokers previous smokers or nonsmokers.

### 2.5. Static Analysis

Descriptive statistics (e.g., percentages, means, standard deviation, SDs) were employed to compare baseline differences among participants, stratified by sex. Cox hazard models were employed to assess the connections between depression, anxiety, and thyroid outcomes. The results were presented as hazard ratios (HRs) along with their corresponding 95% confidence intervals (Cis). Separate models were run for each thyroid outcome: hypothyroidism, hyperthyroidism, NTG, and thyroiditis. To capitalize on existing data to maximize and to mitigate the risk of potential overadjustment of variables that could potentially affect the association between depression, anxiety, and thyroid disorders, Cox hazard models stratified by sex, and adjusted for age and education were conducted. To gauge the dependence of the correlations, HRs were further adjusted based on lifestyle, smoking, and BMI. To establish the relationship between PHQ scores and the risk of thyroid diseases, we also constructed restricted cubic spline analyses.

Statistical analyses employed R software (version 4.0.3, https://www.R-project.org/) for Chi-square tests and Cox hazard model construction, as well as restricted cubic spline analysis. Significance levels were set at 0.05 (two sided). Reporting follows STROBE [35] guidelines.

## 3. Results

**Figure 1:**
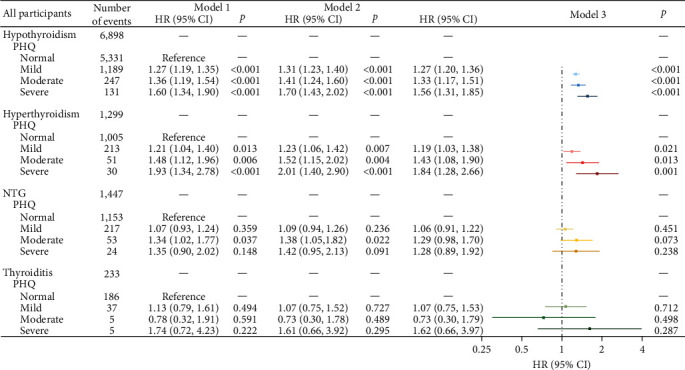
Associations of depression with hypothyroidism, hyperthyroidism, NTG, and thyroiditis. *Note*. HRs, hazard ratios; PHQ, Patient Health Questionnaire; NTG, nontoxic goiter; Ref, reference; CI, confidence interval. Model 1, crude HR; Model 2, adjusted for sex, age, and education; Model 2, adjusted for sex, age, education, smoking, and BMI.

**Figure 2:**
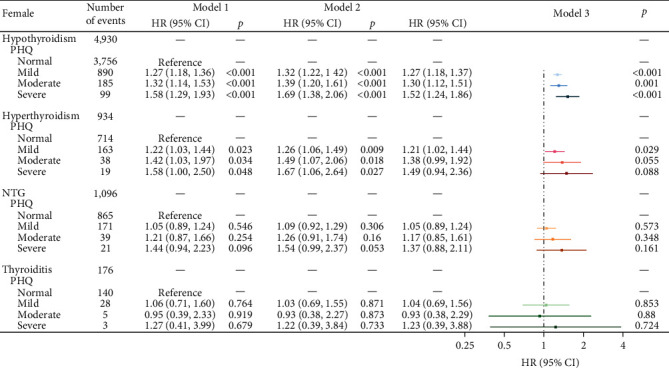
Associations of depression with hypothyroidism, hyperthyroidism, NTG, and thyroiditis in females. Model 1, crude hazard ratios (HRs); Model 2, adjusted for age, and education; Model 3: adjusted for age, education, smoking, and BMI.

**Figure 3:**
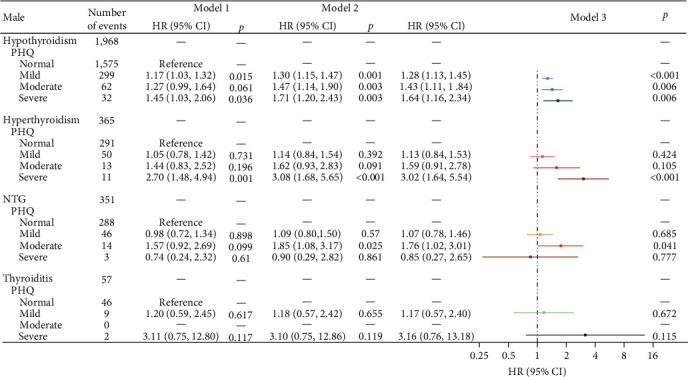
Associations of depression with hypothyroidism, hyperthyroidism, NTG, and thyroiditis in males. Model 1, crude hazard ratios (HRs); Model 2, adjusted for age, and education; Model 3: adjusted for age, education, smoking, and BMI.

**Figure 4:**
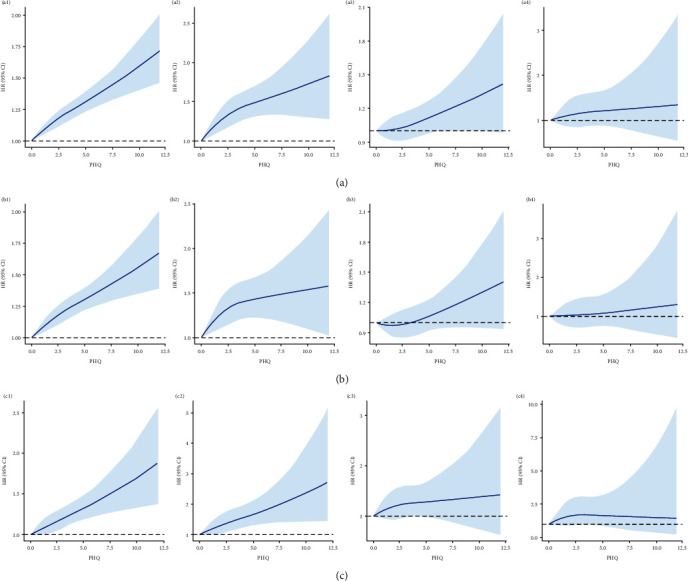
Sex difference in cubic model of the association between depression, hypothyroidism, hyperthyroidism, NTG, and thyroiditis after adjusting for covariates. *Note*. The four images (a1, a2, a3, and a4) in (a) represent all participants, the four images (b1, b2, b3, and b4) in (b) represent female participants, and the four images (c1, c2, c3, and c4) in (c) represent male participants. The order of the images from left to right is hypothyroidism, hyperthyroidism, NTG, and thyroiditis.

As preceding mentioned, a total of 349,993 participants were included, 285,564 (81.6%) do not had anxiety or depression. After stratifying by sex, significant differences were observed between male and female within the sample regarding age, education, smoking, BMI, as well as anxiety and depression (Table 1). During follow-up, 9,877 (2.82%) participants developed thyroid disease (including 6898 hypothyroidism, 1,299 hyperthyroidism, 1,447 NTG, and 233 thyroiditis). When adjusted for sociodemographic factors (sex, age, and education), all levels of depression and anxiety were significantly associated with increased risk of hypothyroidism and hyperthyroidism, after additional adjustments for smoking, and BMI, depression, and anxiety keep significantly associated with increased risk of hypothyroidism (*p*  < 0.05), as the severity of depression and anxiety increased, the risk of illnesses raised, a similar trend is observed in hyperthyroidism. Although NTG and thyroiditis also exhibit this trend, the results are not statistically significant (*p*  > 0.05) (Figure 1). Further, sex-stratified analysis reveals that after adjusting for all the covariates, this trend in hypothyroidism remains significant in both males and females (Figures 2 and 3). In restricted cubic spline analyses, the association between PHQ score and the risk of thyroid disease is shown linear, with the risk increasing with each increment of PHQ score especially in hypothyroidism and hyperthyroidism (Figure 4).

## 4. Discussion

Our study examined the association of depression and anxiety with incident thyroid disease using the UK Biobank population cohort. Among the participants with anxiety and depression in this cohort, the incidence of thyroid disorders was 2.82%, surpassing that of the general population. The high incident risk is especially gathered at the thyroid hormones dysfunctional. Symptoms of depression and anxiety were found to be significantly associated with an increased risk of both hypothyroidism and hyperthyroidism, even after adjusting for various confounding factors, consistent with previous findings [36]. Moreover, the severity of depression and anxiety was positively correlated with the risk of hypothyroidism and hyperthyroidism. Further, gender-stratified analysis revealed that the association between depression/anxiety and hypothyroidism remained significant in both males and females.

Numerous studies have demonstrated an association between depression, anxiety, and thyroid disorders [17, 19, 37, 38]. This relationship has also been observed in certain specific populations. For example, in the elderly population, there is a statistically significant correlation between anxiety and TSH levels in the elderly population [39], besides, fT3/fT4 ratio (fT4, free tetraiodothyronine) is reported to be negatively associated with the severity of depressive symptoms in female adolescents with depression [40]. Despite the high comorbidity and associations that have been demonstrated, there are still some perplexing results, for example, depressive patients with positive thyroid antibodies may be more severe, but they also might have a better treatment response [41].

Anxiety and depression may influence thyroid function through various mechanisms, thereby increasing the risk of thyroid disease. First, the hypothalamic–pituitary–thyroid (HPT) axis plays a significant role in the pathophysiology of depression and anxiety [42]. In patients with depression, the HPT axis is typically inhibited, manifested by an abnormal response of TSH to thyroid-releasing hormone (TRH) [43, 44], this suggests an inadequate response of TSH even in the presence of TRH stimulation in patients with depression. Research indicates that in patients with anxiety disorders, TSH levels tend to be lower, while fT4 levels are higher [45]. Additionally, the responsiveness of TSH to TRH may be diminished in these patients [17]. Similarly, hyperthyroidism and hypothyroidism also exhibit similar hormonal changes, such as reduced sensitivity of TSH to TRH [46]. These findings underscore the association between anxiety and thyroid disorders. Second, depression and anxiety are closely associated with chronic stress states, which contributes to overactivation of the hypothalamic–pituitary–adrenal axis (HPA axis) [47]. Sustained stress responses can lead to elevated cortisol levels [48], which in turn affect the synthesis, secretion, and metabolism processes of thyroid hormone [49], thereby increasing the risk of hypothyroidism. Third, depression and anxiety may also induce autoimmune thyroid diseases such as thyroiditis, by affecting the immune system [50, 51]. The abnormal response of the immune system can lead to damage and inflammation of the thyroid tissue, which in turn affects its normal function [52].

In addition, symptoms of anxiety and depression may lead to changes in behavior and lifestyle, such as irregular diet, lack of exercise, insufficient sleep, etc. [53, 54], what is more, medication usage is another potential factor that cannot be ignored. Selective serotonin reuptake inhibitors have been found to decrease T4 level [55]. It has been reported that tricyclic antidepressants, electroconvulsive therapy, and lithium can also lower T4 level. Carbamazepine also is reported to reduce total T4 levels by decreasing the function of thyroxine-binding globulin [56], all of which may negatively impact thyroid health. This study indicates that in both males and females' patients with hypothyroidism, the severity of depression and anxiety is positively correlated with the risk of hypothyroidism. However, for hyperthyroidism, although a similar trend exists, it is less pronounced in females. Conversely, in males, the risk of developing hyperthyroidism triples when experiencing severe levels of anxiety and depression. This gender disparity may be attributed to physiological and psychological differences in how males and females cope with anxiety and depression [57, 58].

There are several limitations in this study. First, the retrospective self-report questionnaire PHQ-4 used to measure depression and anxiety may cause recall bias, this study evaluates whether depression and anxiety had ever occurred, potential impact of its relief-recurrence fluctuation is not investigated. Second, although thyroid function, such as TSH levels, is an important influencing factor, it has not been included in this study, future research should incorporate data on thyroid function to explore the association between depression and changes in thyroid function. Third, participants in UK Biobank are more likely to be white, affluent, and healthy, this finding may not be generalized to the general population. Fourth, depression and anxiety were assessed using self-report scales. Future research should consider using diagnostic interview tools to better explore the relationship between depression, anxiety, and thyroid disease. However, the UK Biobank made efforts to align its approach with a structured diagnostic framework in accordance with the International Classification of Diseases (ICD-10) and the DSM-IV. Fifth, the absence of information on daily iodine intake in the database could potentially introduce some inaccuracies in the results. Nonetheless, a review of pertinent literature has indicated that the surveyed UK region is not characterized by iodine deficiency [59]. Finally, examining the intake of antidepressant medication and medication that may affect thyroid function such as carbamazepine is important, future research should investigate the effects of these medications.

This study examined the association of depression and anxiety with incident risk of thyroid disorders among middle- and old-aged UK population using the cohort data in UK Biobank. In this large scale, population-based prospective cohort study, we found a higher risk of hypothyroidism and hyperthyroidism among those with anxiety and depression, and highest risk at a severe level. Furthermore, age, sex, smoking, and BMI may potentially influence these associations, further research should be conducted to investigate their underlying biological mechanisms. This implies a relative high risk of developing thyroid disease for individuals with depression and anxiety. Hence, any degree of depression and anxiety should be considered in thyroid disease risk prediction and prevention.

## Figures and Tables

**Table 1:** Baseline characteristics of overall participants, baseline characteristics stratified by sex and group differences in the UK Biobank.

Characteristics	Overall *n* = 349,993	Female *n* = 182,862	Male *n* = 167,131	*p*
Age (years) mean (SD)	55.51 ± 8.08	55.15 ± 7.98	55.90 ± 8.17	<0.001
Education (%)				
College or university degree	142,326 (40.7)	71,733 (39.2)	70,593 (42.2)	<0.001
A levels/AS levels or equivalent	47,866 (13.7)	26,665 (14.6)	21,201 (12.7)	—
O levels/GCSEs or equivalent	88,521 (25.3)	51,045 (27.9)	37,476 (22.4)	—
CSEs or equivalent	22,042 (6.3)	11,428 (6.2)	10,614 (6.4)	—
NVQ or HND or HNC or equivalent	27,615 (7.9)	9,395 (5.1)	18,220 (10.9)	—
Other professional qualifications	21,623 (6.2)	12,596 (6.9)	9,027 (5.4)	—
Smoking (%)				
Never	198,916 (56.8)	112,152 (61.3)	86,764 (51.9)	<0.001
Previous	117,649 (33.6)	55,978 (30.6)	61,671 (36.9)	—
Current	33,428 (9.6)	14,732 (8.1)	18,696 (11.2)	—
BMI (kg/m^2^) Mean (SD)	27.13 ± 4.67	26.68 ± 5.06	27.63 ± 4.14	<0.001
PHQ mean (SD)	1.16 ± 1.99	1.23 ± 2.04	1.09 ± 1.93	<0.001
PHQ (%)				
Normal	285,564 (81.6)	147,209 (80.5)	138,355 (82.8)	<0.001
Mild	50,221(14.4)	27,666 (15.1)	22,555 (13.5)	—
Moderate	9,792 (2.8)	5,508 (3.0)	4,284 (2.6)	—
Severe	4,416 (1.3)	2,479 (1.4)	1,937 (1.2)	—

*Note*. BMI, body mass index; PHQ, score of self-reported Patient Health Questionnaire-4 (PHQ-4).

## Data Availability

The data that support the findings of this study are openly available in UK Biobank, reference number (Application ID: 98111).
